# Chronic Heroin Dependence Leading to Adrenal Insufficiency

**DOI:** 10.1155/2014/461816

**Published:** 2014-08-20

**Authors:** Gautam Das

**Affiliations:** Department of Diabetes and Endocrinology, Prince Charles Hospital, Cwm Taf NHS Trust, Gurnos, Merthyr Tydfil, CF47 9DT, UK

## Abstract

Opioids have been the mainstay for pain relief and palliation over a long period of time. They are commonly abused by drug addicts and such dependence usually imparts severe physiologic effects on multiple organ systems. The negative impact of opioids on the endocrine system is poorly understood and often underestimated. We describe a patient who developed severe suppression of the hypothalamic-pituitary adrenal (HPA) axis leading to secondary adrenal insufficiency due to long standing abuse of opioids.

## 1. Introduction

The long term use of opioids for chronic pain or addiction induces widespread endocrine dysfunction which leads to hypogonadism, infertility, fatigue, depression, anxiety, menstrual irregularities, loss of muscle strength, osteoporosis, and compression fractures. The dynamic functioning of the HPA axis can be modulated by a complex series of exogenous and endogenous influence. Opioids bind to specific receptors in the hypothalamus and pituitary gland, disrupt the pulsatile release of corticotrophin releasing hormone (CRH) and adrenocorticotropic hormone (ACTH), and interfere with the production of cortisol and androgen precursors [1]. These patients also have an atypical circadian rhythm due to alteration of the HPA dynamics which contributes further to adrenal insufficiency [2]. Opiates can also mediate a negative impact on the hypothalamic-pituitary gonadal (HPG) axis resulting in hypogonadism [3].

## 2. Clinical Case 

A 35-year-old female patient was referred to the hospital with a random cortisol of 28 nmol/L found on a routine blood test while being investigated for symptoms of tiredness. A detailed history confirmed spells of lethargy, weight loss, and postural dizziness for few months. This was associated with irregular periods, spontaneous galactorrhea with no headaches, or visual symptoms. She has been a heavy user of heroin and mephedrone on a regular basis for four years but discontinued them only 3-4 weeks prior to presenting to her General Practitioner. She was receiving a combination of buprenorphine and naloxone for depression and opioid dependence and was being actively followed up by the mental health team. She also suffered from asthma and used inhalers very infrequently but has never been on steroid preparations on a long term basis.

Her blood pressure was normal with no postural variation. She was clinically euthyroid and euadrenal. Examination of her breasts revealed minimal milky discharge but her visual acuity and field were normal on confrontation perimetry. A thorough systemic examination was entirely normal. Her blood biochemistry suggested normal levels of urea and electrolytes (U/E's), liver function tests, bone profile, glucose, and plasma osmolality [289 mmol/kg (275–295)], prolactin [182 mU/L (100–500)], LH [4.4 IU/L (2.4–13, follicular phase)], FSH [4.4 IU/L (3.5–13, follicular phase)], and IGF-1 [22.5 nmol/L (9–35)]. Her thyroid function (TFTs) showed a subnormal FT4 [10.2 pmol/L (11–25)] with a normal TSH [2.03 mU/L (0.27–4.2)] and her anti-TPO antibodies were negative [19.4 u/mL (<34)]. A urine toxicology screen confirmed the presence of buprenorphine and benzodiazepines only.

A short synacthen test confirmed adrenal insufficiency with a basal cortisol level of 12 nmol/L and a 30-minute increment to 68 nmol/L (normal > 550 nmol/L) only. Her serum ACTH was low [2.1 pmol/L (4–18)] and the adrenal antibodies were negative. An MRI of pituitary gland was unremarkable. She was commenced on hydrocortisone (20 mg in the morning and 10 mg in the evening) and was followed up in the endocrine clinic. On review, after two months, her symptoms improved remarkably and a repeat biochemistry showed normal U/Es and her TFTs returned to normal (TSH—1.73 mU/L, FT4—11.4 pmol/L) as well.

## 3. Discussion 

Opioid induced endocrinopathy is one of the most common yet least diagnosed and underappreciated consequences of prolonged opioid treatment or abuse. Hypoadrenalism and gonadal steroid depletion have been shown in heroin addicts and in patients on methadone maintenance treatment [3]. The inhibition of the HPA and the HPG axes is mediated by different opioid mechanisms at the hypothalamic level. *δ*- and *κ*-opiate receptors appear to be involved in the control of ACTH release, whereas gonadotrophin secretion is modulated by *ε*-receptors [4]. The impaired pulsatile secretion of CRH due to a variety of putative neurotransmitters and depolarising agents leads to reduced secretion of ACTH and the capacity of the pituitary gland to respond to CRH stimulation [5] which in turn reduces adrenal cortisol production. In addition, there is a dose dependent decrease in adrenal androgen production measured by lowered levels of dehydroepiandrosterone sulphate (DHEAS) [6].

The altered activity of the HPA axis in heroin dependent subjects has been widely studied and is usually characterised by suppressed stress hormone secretion [7]. Injection of diacetylmorphine (DAM) in heroin dependent patients has shown to cause deficit in availability of physiologically active cortisol [8]. Similarly, heroin users also show a decreased HPA activation with metyrapone, a cortisol synthesis inhibitor [9]. Methadone users demonstrate an attenuated response to CRH stimulation and also show a blunted cortisol response to synacthen stimulation [10]. Several individual case reports have also implicated that chronic use of tramadol, fentanyl, and hydromorphone can also lead to adrenocortical insufficiency. Patients with dependence usually have chronically low levels of ACTH and cortisol concentrations which impair an individual's ability to respond to physical, emotional, and metabolic stressors leading to increased cardiovascular risk, abnormal mental health, bone related complications, and metabolic alterations.

## 4. Conclusion 

Clinicians should be aware of such crucial side effects of prolonged opioid use on the endocrine system as the relationship is complex and can catastrophically alter the metabolic homeostasis. A greater emphasis on the use of nonopioid formulations, opioid rotations, and calibrated opioid maintenance therapy should be undertaken in the management of patients with opioid induced endocrine dysfunction.

## Supplementary Material

Supplementary Material: Cortisol, FT3 and FT4 were assayed by electrochemilumincence competitive immunoassay method on Roche MODULAR E170 platform. Hormones like prolactin, FSH, LH, TSH and ACTH were assayed by electrochemilumincence sandwich immunoassay method on Roche MODULAR E170 platform as well. IGF-1 was assayed by Chemilumincence sandwich immunoassay technique on Immuno Diagnosic Systems iSys platform whereas serum osmolality was estimated by freezing point depression method on advanced Instruments osmometer platform.
